# Sample size calculations for cluster randomised controlled trials with a fixed number of clusters

**DOI:** 10.1186/1471-2288-11-102

**Published:** 2011-06-30

**Authors:** Karla Hemming, Alan J Girling, Alice J Sitch, Jennifer Marsh, Richard J Lilford

**Affiliations:** 1 Department of Public Health, Epidemiology and Biostatistics, University of Birmingham, UK

## Abstract

**Abstract:**

**Background**

Cluster randomised controlled trials (CRCTs) are frequently used in health service evaluation. Assuming an average cluster size, required sample sizes are readily computed for both binary and continuous outcomes, by estimating a design effect or inflation factor. However, where the number of clusters are fixed in advance, but where it is possible to increase the number of individuals within each cluster, as is frequently the case in health service evaluation, sample size formulae have been less well studied.

**Methods**

We systematically outline sample size formulae (including required number of randomisation units, detectable difference and power) for CRCTs with a fixed number of clusters, to provide a concise summary for both binary and continuous outcomes. Extensions to the case of unequal cluster sizes are provided.

**Results**

For trials with a fixed number of equal sized clusters (*k*), the trial will be feasible provided the number of clusters is greater than the product of the number of individuals required under individual randomisation (*n_I_*) and the estimated intra-cluster correlation (*ρ*). So, a simple rule is that the number of clusters (*k*) will be sufficient provided:

Where this is not the case, investigators can determine the maximum available power to detect the pre-specified difference, or the minimum detectable difference under the pre-specified value for power.

**Conclusions:**

Designing a CRCT with a fixed number of clusters might mean that the study will not be feasible, leading to the notion of a minimum detectable difference (or a maximum achievable power), irrespective of how many individuals are included within each cluster.

## Introduction

Cluster randomised controlled trials (CRCTs), in which clusters of individuals are randomised to intervention groups, are frequently used in the evaluation of service delivery interventions, primarily to avoid contamination but also for logistic and economic reasons [1-3]. Whilst a well conducted individually Randomised Controlled Trial (RCT) is the gold standard for assessing the effectiveness of pharmacological treatments, the evaluation of many health care service delivery interventions is difficult or impossible without recourse to cluster trials. Standard sample size formulae for CRCTs require the investigator to pre-specify an average cluster size, to determine the number of clusters required. In so doing, these sample size formulae implicitly assume that the number of clusters can be increased as required [1,3-5].

However, when evaluating health care service delivery interventions the number of clusters might be limited to a fixed number even though the sample size within each cluster can be increased. In a real example, evaluating lay pregnancy support workers, clusters consisted of groups of pregnant women under the care of different midwifery teams [6,7]. The available number of clusters was restricted to the midwifery teams within a particular geographical region. Yet within each midwifery team it was possible to recruit any reasonable number of individuals by extending the recruitment period. In another real example, a CRCT to evaluate the effectiveness of a combined polypill (statin, aspirin and blood pressure lowering drugs) in Iran was limited to a fixed number of villages participating in an existing cohort study [8]. Other such examples of designs in which a limited number of clusters were available include trials of community based diabetes educational programs [9] and general practice based interventions to reduce primary care prescribing errors [10], both of which were limited to the number of general practices which agreed to participate.

The existing literature on sample size formulae for CRCTs focuses largely on the case where there is no limit on the number of available clusters [3-5,11,12]. Whilst it is well known that the statistical power that can be achieved by additional recruitment within clusters is limited, and that this depends on the intra-cluster correlation [11-13], little attention has been paid to the limitations imposed when the number of clusters is fixed in advance. This paper aims to fill this gap by exploring the range of effect sizes, and differences between proportions, that can be detected when the number of clusters is fixed. We describe a simple check to determine whether it is feasible to detect a specified effect size (or difference between proportions) when the number of clusters are fixed in advance; and for those cases in which it is infeasible, we determine the minimum detectable difference possible under the required power and the maximum achievable power to detect the required difference. We illustrate these ideas by considering the design of a CRCT to detect an increase in breastfeeding rates where the number of clusters are fixed.

For completeness we outline formulae for simpler designs for which the sample size formulae are relatively well known, or easily derived, as an important prelude. In so doing, the simple relationships between the formulae are clear and this allows progressive development to the less simple situation (that of binary detectable difference or power). It is hoped that by developing the formulae in this way the material will be accessible to applied statisticians and more mathematically minded health care researchers. We also provide a set of guidelines useful for investigators when designing trials of this nature.

## Background

Generally, suppose a trial is to be designed to test the null hypothesis *H*_0 _: *μ*_0 _= *μ*_1 _where *μ*_0 _and *μ*_1 _represent the means of some variable in the control and intervention arms respectively; and where it is assumed that var(*μ*_0_) = var(*μ*_1_) = *σ*^2^. Suppose further that there are an equal number of individuals to be randomised to both arms, letting *n *denote the number of individuals per arm and letting *d *denote the difference to be detected such that *d *= *μ*_0 _- *μ*_1_, 1 - *β *denotes the power and *α *the significance level. We limit our consideration to trials with two equal sized parallel arms, with common standard deviation, two-sided test, and assume normality of outcomes and approximate the variance of the difference of two proportions. The sub-script, I (for Individual randomisation), is used throughout to highlight any quantities which are specific to individual randomisation; and likewise the sub-script, C (for Cluster randomisation), is used throughout to highlight any quantities which are specific to cluster randomisation. No subscripts are used to distinguish cluster from individual randomisation for variables which are pre-specified by the user.

### RCT: sample size formulae under individual randomisation

Following standard formulae, for a trial using individual randomisation[14], for fixed power (1 - *β*) and fixed sample size (*n*) per arm, the detectable difference, *d_I_*, with variance var(*d_I_*) = 2*σ*^2^*/n_I _*is:(1)

where *z*_*α*/2 _denotes the upper 100*α*/2 standard normal centile.

For a trial with *n *individuals per arm, the power to detect a pre-specified difference of *d*, is 1 - *β_I_*, such that:

or equivalently:(2)

where Φ is the cumulative standardised Normal distribution.

And, finally the required sample size per arm for a trial at pre-specified power 1 - *β *to detect a pre-specified difference of *d*, is *n_I_*, where:(3)

Using Normal approximations, the above formulae can be used for binary outcomes, by approximating the variance (*σ*^2^) of the proportions *π*_1 _and *π*_2_, by:(4)

for testing the two sided hypothesis *H*_0 _: *π*_1 _= *π*_2_.

### CRCTs: standard sample size formulae under cluster randomisation

Suppose, instead of randomising over individuals, the trial will randomise the intervention over *k *clusters per arm each of size *m*, to provide a total of *n_C _*= *mk *individuals per arm. Then, by standard results [1], the variance of the difference to be detected *d_C _*is inflated by the Variance Inflation Factor (VIF):(5)

where *ρ *is the Intra-Cluster Correlation (ICC) coefficient, which represents how strongly individuals within clusters are related to each other. Where the cluster sizes are unequal this variance inflation factor can be approximated by:(6)

where *cv *represents the coefficient of variation of the cluster sizes and  is the average cluster size [15]. Thus, the variance of *d_C _*(for fixed cluster sizes) becomes:(7)

and this is simply extended for varying cluster sizes using equation 6. To determine the required sample size for a CRCT with a pre-specified power 1 - *β*, to detect the pre-specified difference *d*, and where there are *m *individuals within each cluster, then the required sample size *n_C _*= *km *per arm, follows straightforwardly from equations 3 and 5 and is:(8)

where *n_I _*is the required sample size per arm using a trial with individual randomization to detect a difference *d*, and VIF can be modified to allow for variation in cluster sizes (equation 6). This is the standard result, that the required sample size for a CRCT is that required under individual randomisation, inflated by the variance inflation factor [1]. The number of clusters required per arm is then:(9)

assuming equal cluster sizes. This slight modification of the common formula for the number of required clusters (over that say presented in [2]), has rounded up the total sample size to a multiple of the cluster size (using the ceiling function). For, unequal cluster sizes (using the VIF at equation 6) this becomes:(10)

again with rounding up to the average cluster size.

## CRCTs of fixed size: fixed number of clusters each of fixed size

Where a CRCT is to be designed with a completely fixed size, that is with a fixed number of clusters, each of a fixed size (although this size may vary between clusters), then it is possible to evaluate both the detectable difference and the power, as would be the case in a design using individual randomisation. CRCTs of fixed size might not be the commonest of designs, but formulae presented below: are an important prelude to later formulae, might be useful for retrospectively computing power once a trial has commenced (and thus the size has been determined), and will also be useful in those limited number of studies for which the trial sample size is indeed completely fixed (for example within a cohort study) [9,10].

### CRCT of fixed size: detectable difference

For a CRCT with a fixed number of clusters *k *per arm, with a fixed number of individuals per cluster *m *and with power 1 -*β*, then the detectable difference, *d_C_*, follows straightforwardly from equation 1:(11)

where *d_I _*is the detectable difference using individual randomisation and VIF might be either of those presented at equations 5 and 6. So the detectable difference in a CRCT can be thought of as the detectable difference in a trial using individual randomisation, inflated by the square-root of the variance inflation factor.

### CRCTs of fixed size: power

The power 1 - *β_C _*of a trial designed to detect a difference of *d *with fixed sample size *n_C _*= *mk *per arm, following equation 2, is:

or equivalently, that:(12)

where again, VIF might be either of those presented at equations 5 and 6. So, power in a CRCT can be thought of as the power available under individual randomisation for a standardised effect size which is deflated by the square-root of the variance inflation factor.

## CRCTs with fixed number of clusters but flexible cluster size

Standard sample size formulae for CRCTs, by assuming knowledge of the cluster size (*m*) and determining the required number of clusters (*k*), implicitly assume that the number of clusters can be increased as required. However, in the design of health service interventions, it is often the case that the number of clusters will be limited by the number of cluster units willing or able to participate. So for example, in two general practice based CRCTs (one to evaluate lay education in diabetes and the other to evaluate a general practice-based intervention to reduce primary care prescribing errors), the number of clusters was limited to the number of primary care practices that agreed to participate in the study. From an estimate of the number of clusters available, it is relatively straightforward to determine the required cluster size for each of the clusters. However, due to the limited increase in precision available by increasing cluster sizes, it might not always be feasible to detect the required difference at required power under a design with a fixed number of *k *clusters. These issues are explored below.

### CRCTs with a fixed number of clusters: sample size per cluster

The standard sample size formulae for CRCTs assumes knowledge of cluster size (*m*) and consequently determines the number of clusters (*k*) required. For a pre-specified available number of clusters (*k*), investigators need instead to determine the required cluster size (*m*). Whilst this sample size formula is not commonly presented in the literature, it consists of a simple re-arrangement of the above formulae presented at equation 8 [2]. So, for a trial with a fixed number of equal sized clusters (*k*) the required sample size per arm for a trial with pre-specified power 1 - *β*, to detect a difference of *d*, is *n_C_*, such that:(13)

where *n_I _*is the sample size required under individual randomisation. This increase in sample size, over that required under individual randomisation, is no longer a simple inflation, as the inflation required is now dependent on the sample size required under individual randomisation.

The corresponding number of individuals in each of the *k *equally sized clusters is:(14)

this time rounding up the total sample size to a multiple of the number of clusters (*k*) available (using the ceiling function).

For unequal cluster sizes, using the VIF from equation 6, the required sample size is:(15)

and the average number of individuals per cluster becomes:(16)

again rounding up to a multiple of the number of clusters (*k*) available.

### CRCT with a fixed number of clusters: feasibility check

When designing a CRCT with a fixed number of clusters, because of the diminishing returns that sets in when the sample size of each cluster is increased, it may not be possible to detect the required difference at pre-specified power [2]. In a CRCT with a fixed number of individuals per cluster, but no limit on the number of clusters, no such limit will exist. This limit on the difference detectable (or alternatively available power) stems from the maximum precision available within a CRCT with a limited number of clusters. Recall that the precision of the estimate of the difference is:(17)

As the cluster size (*m*) becomes large, this precision reaches a theoretical limit:(18)

This limit therefore provides an upper bound on the precision of an estimate from a CRCT. If the CRCT is to achieve the same or greater power as a corresponding individually randomised design, it is required that:(19)

for equal cluster sizes; and:(20)

for unequal cluster sizes. A simple feasibility check, to determine whether a fixed number of available clusters will enable a trial to detect a required difference at required power, therefore consists of evaluating whether the following inequality holds:(21)

for equal cluster sizes [2], and(22)

for unequal cluster sizes. Here, *n_I _*is the required sample size under individual randomisation, *k *is the available number of clusters, *ρ *is the estimated intra-cluster correlation coefficient, and *cv *represents the coefficient of variation of cluster sizes. When this inequality does not hold, it will be necessary to re-evaluate the specifications of this sample size calculation. This might consist of a re-evaluation of the power and significance level of the trial, or it might consist of a re-evaluation of the detectable difference. Bounds, imposed as a result of the limited precision, on the detectable difference and power are derived below.

### CRCT with a fixed number of clusters: minimum detectable difference

For a trial with a fixed number of clusters (*k*), and power 1 - *β*, the theoretical Minimum Detectable Difference (MDD) for an infinite cluster size is *d_MDD_*, where:(23)

which follows naturally from the formula for detectable difference (equation 1) and the bound on precision (equation 18). This therefore gives a bound on the detectable difference achievable in a trial with a fixed number of clusters.

For the case of two binary outcomes, where *π*_1 _is fixed (and *π*_2 _*> π*_1_), then the minimum detectable difference for a fixed number of clusters per arm *k*, is *d_C _*= *π*_2 _- *π*_1 _such that:(24)

Re-arranging this as a function of *π*_2 _is:(25)

where *a *= - (1 + *w*), *b *= 2*π*_1 _+ *w*, , and *w *= *ρ*(*z*_*α*/2 _+ *z*_*β*_)*^2^*/*k*. Solving this quadratic gives:(26)

Each of these two solutions to this quadratic will provide the limit on *π*_2 _for two sided tests.

### CRCT with a fixed number of clusters: maximum achievable power

For a trial again with a fixed number of clusters (*k*), the theoretical Maximum Achievable Power (MAP) to detect a difference *d *is 1 - *β _MAP _*where:(27)

which again follows from the formula for power (equation 2) and the bound on precision (equation 18). So the maximum achievable power is 1 - *β_MAP _*where:(28)

This therefore provides an upper limit on the power available under a design with a fixed number of clusters *k*.

### CRCT with a fixed number of clusters: practical advice

When designing a CRCT with a fixed number of clusters, researchers should be aware that such trials will have a limited available power, even when it is possible to increase the number of individuals per cluster. In such circumstances, it will be necessary to:

(a) Determine the required number of individuals per arm in a trial using individual randomisation (*n_I_*).

(b) Determine whether a sufficient number of clusters are available. For equal sized clusters, this will occur when:

where *n_I _*is the sample size required under individual randomisation, *ρ *is the intra-cluster correlation coefficient, and *k *is the number of clusters available in each arm. For unequal sized clusters:

where *cv *is the coefficient of variation of cluster sizes.

(c) Where the design is not feasible and cluster sizes are unequal, determine whether the design becomes feasible with equal cluster sizes (i.e. if *k > n_I_ρ*).

(d) Where the design is still not feasible:

(i) Either: the power must be reset at a value lower than the maximum available power (equation 28),

(ii) Or: the detectable difference must be set greater than the minimum detectable difference (equations 23 (continuous outcomes) and 26 (binary outcomes)),

(iii) Or: both power and detectable difference are adjusted in combination.

(e) Once a feasible design is found, determine the required number of individuals per cluster from equations 14 (for equal cluster sizes) and 16 (for varying cluster sizes).

### General examples

**Figure 1:**
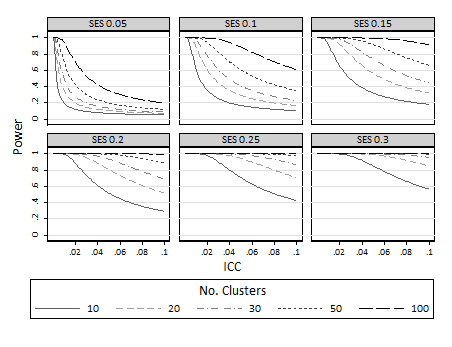
**Maximum achievable power for various different standardised effect sizes: limiting values as the cluster size approaches infinity**.

**Figure 2:**
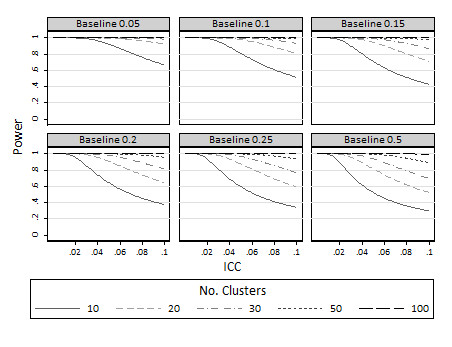
**Maximum achievable power to detect increases in 10 percentage points for various different baseline proportions (*π*_1_): limiting values as the cluster size approaches infinity**.

Maximum achievable power for cluster designs with 10, 20, 30, 50 or 100 clusters per arm are presented in Figure 1 for standardised effect sizes ranging from 0.05 to 0.30 and for ICCs in the range 0 to 0.1 (which are common ICCs in the medical literature [16]). As expected, achievable power increases with increasing numbers of clusters and increasing effect size. For the smallest effect size considered, 0.05, even 100 clusters per arm is not sufficient to obtain anywhere near an acceptable power level for ICCs above about 0.02. For less extreme effect sizes, such as 0.2 when there are 50 or 100 clusters available per arm, for ICCs less than about 0.1 power in the level of 80% will be obtainable; yet where there are just 10 or 20 clusters available, 80% power will only be attainable for ICCs less than about 0.06. Figure 2 shows similar estimates of maximum achievable power for binary comparisons at baseline proportions ranging from 0.05 to 0.5 to detect increases of 0.1 (i.e. 10 percentage points on a percentage scale).

**Figure 3:**
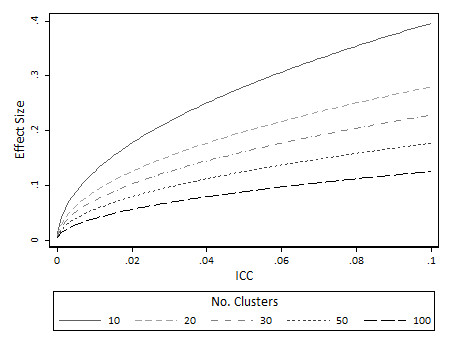
**Minimum detectable difference (effect size) at 80% power for continuous outcomes: limiting values as the cluster size approaches infinity**.

**Figure 4:**
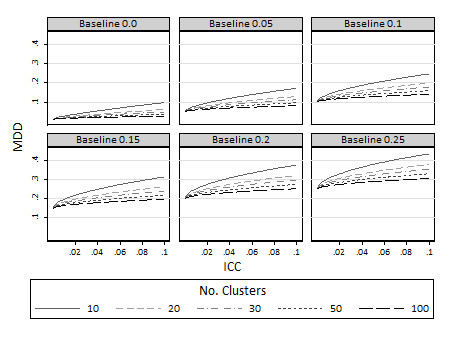
**Minimum detectable difference (*π*_2_) at 80% power various different baseline proportions (*π*_1_): limiting values as the cluster size approaches infinity**.

Minimal detectable differences are also presented for both standardised effect sizes (Figure 3) and proportions (Figure 4) for 80% power. As expected, increasing the number of clusters reduces the minimum detectable difference. Therefore with a large number of clusters available and sufficient numbers of individuals per cluster, trials are possible to detect small changes in proportions and standardised effect sizes. On the other hand, for trials with few clusters (say 10 or 20 per arm), minimum detectable differences become large. So, for example for continuous outcomes, with say 10 clusters per arm and an ICC in the region of 0.02, then the MDD is in the region of 0.2 standardised effect sizes (Figure 3). For binary outcomes (Figure 4) with 10 clusters per arm and ICC in the region of 0.02 the minimum detectable difference is in the region of about a 10 percentage point change (i.e. from about 15% to 25%).

## Example

In a real example, a CRCT is to be designed to evaluate the effectiveness of lay support workers to promote breastfeeding initiation and sustainability until 6 weeks postpartum. Due to fears of contamination, whereby new mothers indivertibly gain access and support from the lay workers, the intervention is to be randomised over cluster units. Cluster randomisation will also ensure that the trial is logistically simpler to run, as randomisation will be carried out at a single point in time, and midwives will have the benefit of remaining in either the intervention or control arm for the duration of the trial. The cluster units to be used are midwifery teams, which are teams of midwives who visit a set number of primary care general practices to deliver antenatal and postnatal care. The trial is to be carried out within a single primary care trust within the West Midlands. The nature of this design therefore means that the number of clusters available is fixed at the number of midwifery teams delivering care within the region.

At the time of designing the trial, current breastfeeding rates, at 6 weeks postpartum, in the region were around 40%. National targets had been set to encourage all regions to increase rates to around 50%. It was known that 40 clusters are available (i.e. there are 40 midwifery teams within the region), so that the number of clusters per arm was fixed to *k *= 20. Estimates of ICC range from 0.005 to 0.07 in similar trials [6,7].

Firstly, the feasibility check is implemented to determine whether the 20 available clusters per arm are sufficient to detect the 10 percentage point change assuming the lower estimated ICC (0.005). Where the power is set at 80%, the required sample size per arm to detect an increase in percentages from 40% to 50%, under individual randomisation, is *n_I _*= 385 (Table 1). When multiplied by the ICC this gives 385 × 0.005 = 1.925 which is less than *k *= 20. This therefore means that 20 clusters per arm will be sufficient for this design (provided an adequate number of individuals are recruited in each cluster). A similar design with 90% power would require 519 individuals per arm using individual randomisation. Again, because 515 × 0.005 = 2.57 < 20, this also means that 20 clusters per arm will be sufficient to detect an increase from 40% to 50% with 90% power (again provided an adequate number of individuals are recruited in each cluster). Equation 14 shows that under the assumption that *ρ *= 0.005, either 22 or 30 individuals will be required per cluster (for 80% and 90% power respectively).

**Table 1:** Estimates of the Minimum Detectable Difference (MDD) for trial with 20 clusters per arm, to detect an increase in an event rate from 40%

	Power = 80%		Power = 90%	
	**40% vs 50%**	**MDD**	**40% vs 50%**	**MDD**

ICC = 0.005	*n_I _*= 385	N/A	*n_I _*= 515	N/A
	*n_C _*= 440		*n_C _*= 600	
	m = 22		m = 30	

ICC = 0.07		MDD = 12%		MDD = 14%
		*n_I _*= 267		*n_I _*= 262
		*n_C _*= 3,780		*n_C _*= 2,920
		m = 189		m = 146

Secondly, the feasibility check is evaluated to determine whether the 20 available clusters per arm is sufficient to detect the 10 percentage point change assuming the higher estimated ICC (0.07). However, in this case as 385 × 0.07 = 26.95 *>*20, so the condition is not met at the 80% power level (and so neither at the 90% power level). Therefore, 20 clusters per arm is not a sufficient number of clusters, however many individuals are included within each cluster, to detect the required effect size at the pre-specified power and significance.

Since this latter design is not feasible, formulae at equation 25 allow determination of the minimum detectable difference (or maximum achievable power from equation 27). For a cluster trial with 80% power, and assuming a baseline event rate of *π*_1 _= 0.40, the minimum detectable difference is 0.12 (to 2 d.p.). That is, a change from 40% to 52%. To detect a change from 40% to 52% with 80% power, 189 individuals would be required per cluster. For a trial with 90% power, the minimum detectable difference is 0.14 (i.e. a change from 40% to 54%). To detect a change from 40% to 54% with 90% power, 146 individuals would be required per cluster.

## Discussion

In health care service evaluation cluster RCTs, pre-specifying the numbers of clusters available, are frequently used. That is, trials are designed based on a limited number of cluster units (e.g. GP practices) willing or able to participate [6,7,9,10]. In contrast, sample size methods are almost exclusively based on pre-specified average cluster sizes, as opposed to number of clusters available [1,4]. Whilst mapping sample size formulae from one method to the other is straightforward, a limit on the precision of estimates in such designs leads to a maximum available power (that is, a limit on the power available irrespective of how large the clusters are) and minimum detectable differences (that is, a limit on the difference detectable irrespective of how large the clusters are).

For example, with just 15 clusters available per arm and an ICC of 0.05, power achievable for a trial aiming to detect an increase in percentage change from 40% to 50% is limited to about 62%, irrespective of how large the clusters are made. Cluster trials with just 15 clusters available per arm are not uncommon and a 10 percentage point change not an unrealistic goal in many settings. However, power levels as low as 60% are clearly sub-optimal, and might not be regarded as sufficiently high to warrant the costs of a clinical trial. Formulae provided here for minimum detectable differences show that to retain a power level in the region of 80%, triallists would have to be content with detecting a difference above a twelve percentage point change. Re-formulation of the problem in terms of minimum detectable difference can thus be used to compare the difference which is statistically detectable (at acceptable power levels) to that which is clinically, or managerially, important.

Should the situation arise in which the postulated ICC suggests that it is not possible to detect the required difference (at pre-specified power), it might be tempting to lower the estimated ICC. Such an approach should be strongly discouraged, since loss of power will most likely result, potentially leading to a non-significant finding [12]. Rather, formulae here allow sensitivity of the design to be explored in light of possible variations in the ICC. However, other avenues to increase available power might reasonably be considered. For example, it may be plausible to consider relaxing alpha and even to set alpha and beta equivalent [17]. Or alternatively, incorporating prior information in a Bayesian framework may lead to increases in power. It might further be argued that studies of limited power are of importance as they contribute to the evidence framework by ultimately becoming part of future systematic reviews [18], and the methods presented here thus allow for the achievable power to be computed. Before-and-after type studies offer a further avenue of exploration, as by their very nature induce smaller intra-cluster correlations.

Methodological limitations of the work presented here include the assumption of equal sized arms; equal standard deviations; Normality assumptions (which might not be tenable for small numbers of clusters as well as small numbers of individuals); and lack of continuity correction for binary variables. Furthermore, CRCTs with a small number of clusters are controversial, primarily because the small number of units randomised open results to the possibility of bias and approximations to Normality become questionable. However, despite this, CRCTs with a small number of clusters are frequently reported. The Medical Research Council, for instance, has issued guidelines that cluster trials with fewer than 5 clusters per arm are inadvisable [19]. Others have considered some of the issues involved in community based intervention trials with a small number of clusters, but have focused on issues of restricted randomisation and whether the analysis should be at the individual or cluster level [20].

## Conclusions

Evaluations of health service interventions using CRCTs, are frequently designed with a limited available number of clusters. Sample size formulae for CRCTs, are almost exclusively evaluated as a function of the average cluster size. Where no formal limits exist on the number of individuals enrolled within each cluster, increasing the numbers of individuals leads to a limited increase in the study power. This in turn means that for a trial with a fixed number of clusters, some designs will not be feasible, and we have provided simple guidelines to evaluate feasibility. A simple rule is that the number of clusters (*k*) will be sufficient provided:

For infeasible designs to retain acceptable levels of power, detectable difference might not be as small as desired, leading to the notion of a minimum detectable difference. Useful aidese memoires are that the detectable difference in a CRCT is that of an individual RCT inflated by the square root of the variance inflation factor; and the power is that under individual randomisation with the standardised effect size deflated by the square root of the variance inflation factor. A STATA function, clusterSampleSize.ado, allows practical implementation of all formulae discussed here and is available from the author.

## Competing interests

The authors declare that they have no competing interests.

## Authors' contributions

KH, JM and AS conceived the idea. KH wrote the first and subsequent drafts. AG and RJL helped developed the ideas. All authors read and approved the final manuscript.

## Pre-publication history

The pre-publication history for this paper can be accessed here:

http://www.biomedcentral.com/1471-2288/11/102/prepub
