# Erratum to: Sample size calculations for cluster randomised controlled trials with a fixed number of clusters

**DOI:** 10.1186/s12874-017-0292-x

**Published:** 2017-01-19

**Authors:** Karla Hemming, Alan J Girling, Alice J Sitch, Jennifer Marsh, Richard J Lilford

**Affiliations:** Department of Public Health, Epidemiology and Biostatistics, University of Birmingham, Birmingham, UK

## Erratum

After publication of the original article [1], it came to the authors’ attention that there was a typing error in equation 16. There should be a rho on the denominator of this equation.

The published version of equation 16 is:

$$ m=\frac{\left(1-\rho \right){n}_{I\ }}{k-{n}_I\left(c{v}^2+1\right)} $$

The correct version of equation 16 is:

$$ m=\frac{\left(1-\rho \right){n}_{I\ }}{k-{n}_I\left(c{v}^2+1\right)\rho } $$
